# *Candida*-Reactive T Cells for the Diagnosis of Invasive *Candida* Infection—A Prospective Pilot Study

**DOI:** 10.3389/fmicb.2018.01381

**Published:** 2018-06-22

**Authors:** Felix C. Koehler, Oliver A. Cornely, Hilmar Wisplinghoff, Astrid C. Schauss, Jon Salmanton-Garcia, Helmut Ostermann, Maren Ziegler, Petra Bacher, Alexander Scheffold, Regina Alex, Anne Richter, Philipp Koehler

**Affiliations:** ^1^Cologne Excellence Cluster on Cellular Stress Responses in Aging-Associated Diseases, University of Cologne, Cologne, Germany; ^2^Department I of Internal Medicine, ECMM Diamond Center of Excellence in Medical Mycology, German Centre for Infection Research (DZIF), University of Cologne, Cologne, Germany; ^3^Clinical Trials Centre Cologne (ZKS Köln), University of Cologne, Cologne, Germany; ^4^Labor Dr. Wisplinghoff, Cologne, Germany; ^5^Institute for Medical Microbiology, Immunology and Hygiene, University of Cologne, Cologne, Germany; ^6^Institute for Clinical Microbiology, University Witten/Herdecke, Witten, Germany; ^7^Department of Internal Medicine III, University of Munich, Munich, Germany; ^8^Department of Cellular Immunology, Clinic for Rheumatology and Clinical Immunology, Charité–University Medicine Berlin, Berlin, Germany; ^9^German Rheumatism Research Centre (DRFZ) and Leibniz Association, Berlin, Germany; ^10^Miltenyi Biotec GmbH, Bergisch Gladbach, Germany

**Keywords:** invasive candidiasis, candidemia, hepatosplenic candidiasis, flow cytometry, fungus-reactive T cells, CD154

## Abstract

**Background:** Blood or tissue culture or histology prove invasive *Candida* infection, but long time to result, limited feasibility and sensitivity call for new approaches. In this pilot project, we describe the diagnostic potential of quantitating *Candida*-reactive, CD4/CD69/CD154 positive lymphocytes in blood of patients with invasive *Candida* infection.

**Methods:** We used flow cytometry quantitating *Candida*-reactive, CD4/CD69/CD154 positive lymphocytes from peripheral blood of patients with invasive *Candida* infection, from patients at risk and healthy volunteers as controls.

**Results:** Elevated levels of *Candida*-reactive lymphocytes were measured in 13 patients with proven invasive *Candida* infection and in one patient with probable hepatosplenic candidiasis. Results of three candidemia patients were uninterpretable due to autofluorescence of samples. Twelve of 13 patients had *Candida* identified to species level by conventional methods, and T cell reactivity correctly identified *Candida* species in 10 of 12 patients. Nine hematological high-risk patients and 14 healthy donors had no elevated *Candida*-reactive T cell counts.

**Conclusions:** This *Candida*-reactive lymphocyte assay correctly identified the majority of patients with invasive *Candida* infection and the respective species. Our assay has the potential to support diagnosis of invasive *Candida* infection to species level and to facilitate tailored treatment even when biopsies are contraindicated or cultures remain negative.

## Introduction

Invasive *Candida* Infection (ICI) is among the most common bloodstream infections and represents the most common invasive fungal infection (Wisplinghoff et al., 2004; Kullberg and Arendrup, 2015). *Candida* spp. are part of the mucosal mycobiota, and may translocate to the bloodstream. *Candida* spp. may also cause complicated deep tissue disease. Organs frequently involved are kidney, spleen, liver, central nervous system, eyes, bones and joints (Arendrup, 2010; Kullberg and Arendrup, 2015).

ICI is proven by direct tests, such as culture, histopathology or direct microscopy from biopsy samples (Cuenca-Estrella et al., 2012). Blood culture sensitivity is reported with 21–71% (Ness et al., 1989; Kami et al., 2002). Underlying medical conditions often prevent biopsies (Clancy and Nguyen, 2013). Indirect tests, such as *Candida* mannan antigen, anti-mannan antibodies, *Candida albicans* germ tube gem tube antibodies (CAGTA) and β-1,3-D-glucan (BDG) are not species specific (Ponton et al., 1994; Laín et al., 2007; Mikulska et al., 2010; Lamoth et al., 2012). Polymerase chain reaction (PCR) based assays are being evaluated, but lack validation and standardization in addition to variable sensitivity (Schelenz et al., 2015; Pappas et al., 2016).

Fungal antigens activate CD4^+^ T cells, which initiate inflammatory and antifungal immune response (Romani, 2011). These fungal-reactive T cells are specifically directed to individual pathogens and may promise species specificity (Bacher et al., 2013).

We used a recently developed flow cytometry assay quantitating *Candida*-reactive CD4^+^ T cells for ICI patients identifying *Candida* to species level (Bacher et al., 2015). The basic principle is the stimulation of patient derived CD4^+^ T cells with lysates from either *C. albicans, C. glabrata, C. parapsilosis, C. tropicalis, or C. krusei*. *Candida*-spp.-reactive CD4^+^ T cells upregulate the activation markers CD154 (CD40L) and CD69, which are detected by flow cytometry (Frentsch et al., 2005; Bacher et al., 2013, 2014, 2015; Cossarizza et al., 2017). In a single case report of a *Candida* spondylodiscitis we previously demonstrated the benefits of the *Candida*-reactive lymphocyte assay detecting ICI and *Candida* spp. allowing tailored treatment (Koehler et al., 2017).

## Material and methods

### Mechanical lysis of *candida* spp.

*C. albicans, C. glabrata, C. parapsilosis, C. tropicalis*, and *C. krusei*. were cultured for 5 days at 37°C in Sabouraud 2% Glucose Media (Carl Roth, Karlsruhe, Germany). *Candida* cells were recovered by centrifugation and washed with ultra-pure water (Biochrom GmbH, Berlin, Germany). For total mycelial lysate *Candida* cells were suspended in Dulbecco's PBS (PromoCell; Heidelberg, Germany) and lysed mechanically using gentleMACS^TM^ M tubes and gentleMACS^TM^ Dissociator (both Miltenyi Biotec GmbH, Bergisch Gladbach, Germany). Lysed mycelium of each *Candida* spp. was collected by centrifugation.

By use of Pierce^TM^ BCA Protein Assay Kit (Themo Fisher Scientific, Waltham, MA, USA) the concentration of *Candida* lysates was determined.

Lysates of each *Candida* spp. were stored separately in aliquots at −80°C.

### Study participants

Adult patients at risk were eligible to participate to the survey per protocol – Improving Diagnosis of Severe Infections of Immunocompromised Patients (ISI) (Identifier of the University of Cologne Ethics Committee: 08-160). Written informed consent was obtained from each patient or the legal guardian.

Buffy coats or peripheral ethylenediaminetetraacetic acid (EDTA) blood samples were obtained from the Institute for Transfusion Medicine, Klinikum Dortmund gGmbH, Germany, the DRK Dresden, Germany, the Charité blood bank, Charité Berlin, Germany or from in-house volunteers after informed consent (Identifiers of the Charité Berlin Ethics Committee: EA1/149/12; EA1/272/15).

### Cell preparation

Peripheral blood mononuclear cells (PBMC) were isolated from EDTA blood samples by density centrifugation with density gradient media (Axis-Shield, Oslo, Norway). PBMCs were washed with CliniMACS PBS/EDTA buffer and RPMI-1640 medium and were re-suspended with RPMI-1640 medium supplemented with 5% Gemcell^TM^ human AB serum (Gemini Bio Products, West Sacramento, CA, USA) and 1% 2 mM L-glutamin (Lonza Group, Basel, Switzerland).

### Detection of viable cells and culture of PBMCs

To quantify viable cell count, PBMCs were diluted 1:10 in RPMI-1640 medium and stained with 7-aminoactinomycin D (7AAD) (Miltenyi Biotec GmbH and eBioscience, San Diego, CA, USA). Data were measured with a MACSQuant® Analyzer 10 and MACSQuantify^TM^ software (Version 2.6, both Miltenyi Biotec GmbH,) was used for evaluation. 1 × 10^6^ cells/100 μl and well were seeded in 100 μl RPMI-1640 medium supplemented with 5% Gemcell^TM^ human AB serum and 1% 2 mM L-Glutamin into 96 well flat-bottom plates. PBMCs were incubated overnight at 37°C with 5% CO_2_.

### Stimulation of PBMCs

**Figure 1:**
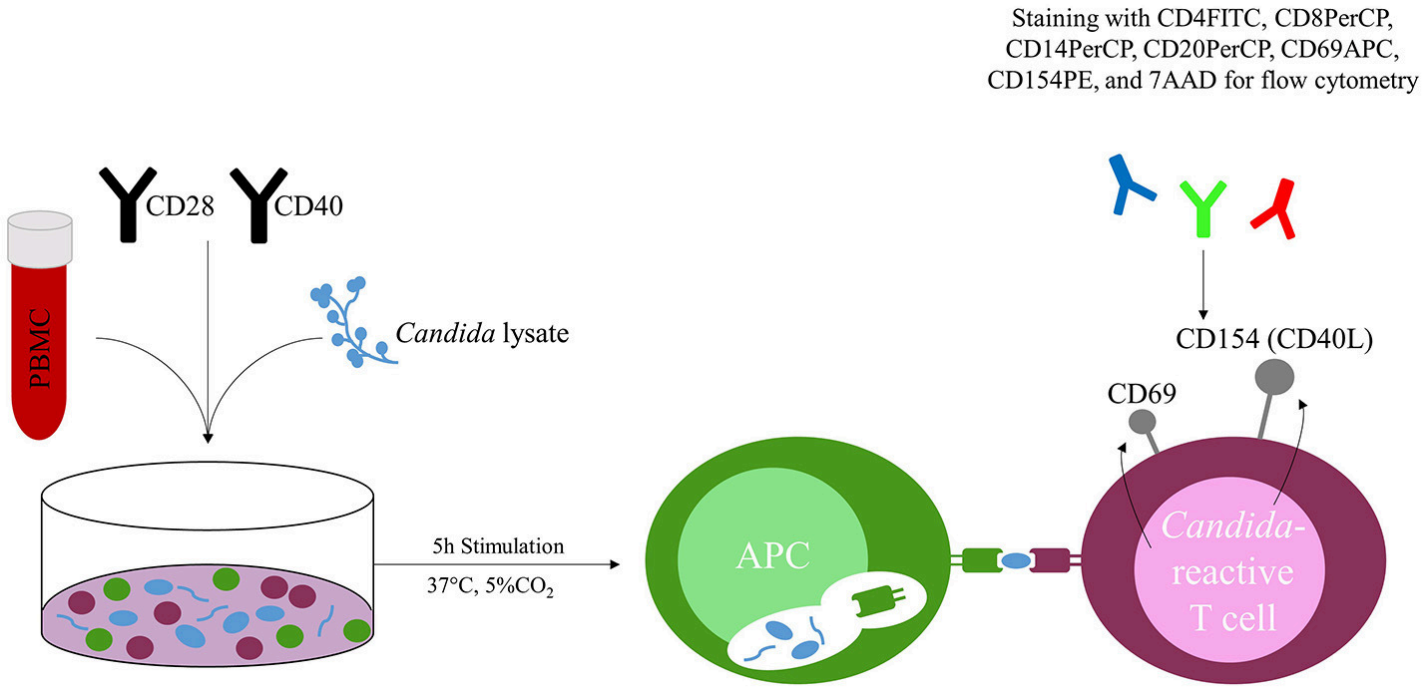
Concept of the *Candida*-reactive lymphocyte assay. Peripheral blood mononuclear cells (PBMC) are co-incubated with *Candida* spp. specific lysate (e.g., *C. albicans*) and stimulated with CD28 and CD40 antibodies. T cells react to the presented fungal peptides on the surface of antigen presenting cells (APC) and upregulate the activation markers CD69 and CD154 (CD40L), which are quantified by flow cytometry after 5 h stimulation at 37°C and 5% CO_2_.

Cultured cells were stimulated with CD28 and CD40 pure antibodies (Miltenyi Biotec GmbH) and with lysate of *C. albicans, C. glabrata, C. parapsilosis, C. tropicalis, C. krusei* or Staphylococcal Enterotoxin B (SEB) (1 μg ml^−1^; Sigma-Aldrich GmbH, Munich, Germany) for 5 h at 37°C with 5% CO_2_. (Figure 1) Each *Candida* species was tested in separate stimulation. Missing challenge with fungal lysate served as negative control, addition of SEB as positive control.

### Flow cytometry

**Figure 2:**
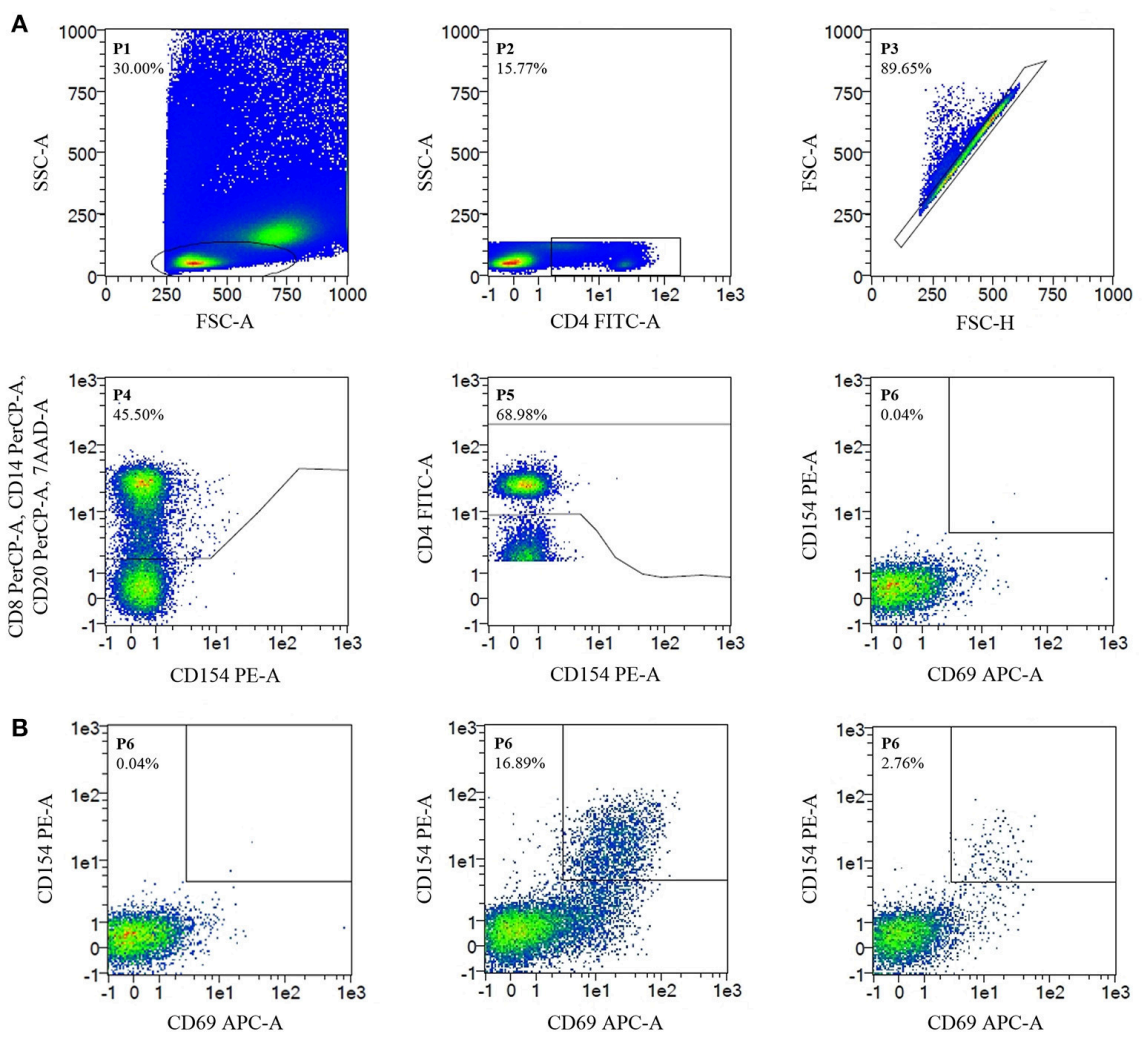
Flow Cytometry-Gating strategy and detection of *C. glabrata*-reactive T cells. Cell frequencies (%). **(A)** Gating strategy. Negative control, unstimulated CD4^+^ T cells, show no CD69/CD154 expression. **(B)** Detection of *C. glabrata*-reactive T cells. From left to right. Negative control; positive control (staphylococcal enterotoxin B stimulated CD4^+^ T cells show CD69/CD154 double-expression); antigen-stimulated probe (CD69/CD154 double expression of *C. glabrata*-reactive CD4^+^ T cells after antigen stimulation with *C. glabrata* lysate).

Stimulated PBMC were stained with CD4FITC (VIT4), CD8PerCP (BW135/80), CD14PerCP (TÜK4), CD20PerCP (LT20), CD69APC (FN50), CD154PE (5C8) (all Miltenyi Biotec GmbH) and 7AAD (Figure 1). Data were acquired on a MACSQuant® Analyzer 10 and MACSQuantify^TM^ software was used for analysis. *Candida*-reactive CD4^+^ T cells were detected based on the upregulation of CD69 and CD154 (CD40L) (Figure 1). Gating strategy is shown in Figure 2.

### Fluorescence microscopy

For fluorescence microscopy stimulated PBMCs were stained with CD4FITC (VIT4), CD69APC (FN50), and CD154PE (5C8) and microscopy was performed with a Zeiss LSM Meta710 confocal microscope (Carl Zeiss Microscopy GmbH, Jena, Germany).

### Conventional diagnostics, study design and definitions

Blood-cultures were processed using the BactAlert 3D (BioMerieux, Marcy l'etoile France) and the Bactec (BectonDickinson, Franklin Lakes, NJ, USA) systems. Identification (ID) of *Candida* spp. to species level and susceptibility testing (AST) was performed using VITEK2 (ID and AST) and API (ID, both, BioMerieux) and the MALDI Biotyper® (ID, Bruker Daltonics, Hilden, Germany), and E-Test (AST, BioMerieux). BDG measurements were done using the Fungitell ß-D-glucan ELISA assay (Associates of Cape Cod, MA, USA). Histology was stained with Grocott methenamine silver staining (Merck, Darmstadt, Germany and Diagonal GmbH, Münster, Germany).

To examine the performance of *Candida*-reactive T cells as diagnostic read-out for ICI, patients with proven and suspected ICI and disease control patients were enrolled in the web-based database of the European Confederation of Medical Mycology (ECMM) *Candida* Registry *Candi*Reg (Identifier of the University of Cologne Ethics Committee: 17-485, ClinicalTrials.gov Identifier: NCT03450005) (ECMM, 2018). Risk factors, site of infection, symptoms, diagnosis, treatment and outcome were documented.

For clinical evaluation patients cohorts were grouped according to the 2008 European Organization for Research and Treatment of Cancer/Invasive Fungal Infections Cooperative Group and the National Institute of Allergy and Infectious Diseases Mycoses Study Group (EORTC/MSG) criteria (De Pauw et al., 2008).

### Statistical analysis

Cut-off values discriminating between healthy donors, disease control and patients with proven ICI were calculated by receiver operating characteristic (ROC) analysis and *p*-values were determined by Fisher's exact test using IBM SPSS Statistics software (Version 23, IBM Corporation, Armonk, NY, USA). For the determination of the cut-off value of fold increase of antigen-stimulated T cells compared to unstimulated T cells, we excluded measurements of proven ICI patients in the presence of antifungal treatment without elevated frequencies of *Candida*-reactive T cells. To examine the cut-offs of levels of *Candida*-reactive T cells discriminating between individuals with and without ICI we excluded measurements of proven ICI patients in the presence of antifungal treatment without elevated frequencies of *Candida*-reactive T cells. Measurements of proven ICI patients with a fold frequencies smaller than the calculated 3.05-fold increase were excluded to exclude false-positive results and to find correct and sufficient stimulations.

## Results

### Patient characteristics

We determined the performance of the *Candida*-reactive lymphocyte assay in a cohort of 26 patients. Three (11.5%) patients were excluded from analysis due to autofluorescence of cells leading to elevated background expression of CD69^+^/CD154^+^ T cells. Thirteen patients (56.5%) had proven and one patient (4.3%) probable ICI, a culture negative hepatosplenic candidiasis (De Pauw et al., 2008). Nine hematological high-risk patients (39.1%) served as disease control cohort. To examine the mean frequency of *C. albicans*-reactive T cells in healthy individuals we included an additional cohort of 96 healthy blood donors.

Patient characteristics and demographic data are given in Table 1. Most common underlying conditions of patients with proven or probable ICI were hematological and oncological malignancies (*n* = 11, 78.4%). More than half of the patients were treated on intensive care unit (ICU) prior the diagnosis of ICI (*n* = 8, 57.1%). Eight patients (57.1%) received chemotherapy within 3 months prior the diagnosis of ICI and five patients (35.7%) had undergone major surgery recently. Most prevalent site of infection was the blood stream in 12 patients (85.7%). Hepatosplenic candidiasis was found in three patients (21.4%). Disseminated candidiasis, defined as positive blood culture and/or at least two non-contiguous sites affected, was diagnosed in four patients (28.6%).

**Table 1:** Baseline characteristics of study participants.

**Variables**	**Proven and probable ICI (*n* = 14)**	**Disease control (*n* = 9)**
**AGE, YRS**.		
Median and SD	64.0 ± 14.1	70.0 ± 14.1
Range	33–78	34–75
**BMI, kg/m**^2^		
Median and SD	22.6 ± 7.3	24.8 ± 6.8
Range	12.1–40.1	13.2–38.4
**GENDER**, ***n*** **(%)**		
Female	6 (42.9)	6 (66.7)
**ETHNIC ORIGIN**, ***n*** **(%)**		
Caucasian (White)	13 (92.9)	9 (100.0)
Unknown	1 (7.1)	
**RISK FACTORS**, ***n*** (%)^*^		
Chemotherapy	8 (57.1)	6 (66.7)
Hematopoietic stem cell transplantation (HSCT)		3 (33.3)
Radiotherapy	2 (14.3)	1 (11.1)
Neutropenia	3 (21.4)	5 (55.6)
Surgery	5 (35.7)	2 (22.2)
**UNDERLYING CONDITIONS**, ***n*** (%)^*^		
Hematological/Oncological malignancy	11 (78.4)	8 (88.9)
HIV/AIDS	2 (14.3)	
Solid organ transplantation		1 (11.1)
Rheumatic diseases/Autoimmune disorder	1 (7.1)	2 (22.2)
Chronic cardiovascular disease	8 (57.1)	3 (33.3)
Chronic liver disease	3 (21.4)	
Chronic pulmonary disease		1 (11.1)
Chronic renal disease	2 (14.3)	1 (11.1)
Diabetes mellitus	5 (35.7)	4 (44.4)
Viral pneumonia	1 (7.1)	1 (11.1)
Alcohol addiction	2 (14.3)	
Obesity or underweight^†^	6 (42.9)	2 (22.2)
ICU treatment	8 (57.1)	4 (44.4)
**SIGNS AND SYMPTOMS**, ***n*** (%)^*^		
Fever	7 (50.0)	
Chills	3 (21.4)	
Tachycardia	2 (14.3)	
Tachypnea	2 (14.3)	
Heart failure	2 (14.3)	
Hepatosplenomegaly	1 (7.1)	1 (11.1)
**SITE OF INFECTION**, ***n*** (%)^*^		
Blood (culture positive)	12 (85.7)	
Liver	3 (21.4)	
Spleen	3 (21.4)	
Peritoneum	1 (7.1)	
Bones and joints	1 (7.1)	
Eye	1 (7.1)	
Foreign bodies	3 (21.4)	
Disseminated^**^	4 (28.6)	

*AIDS, acquired immunodeficiency syndrome; BMI, body mass index; CNS, central nervous system; HIV, human immunodeficiency virus; ICU, intensive care unit; SD, standard deviation*;

* >1 factor possible per patient;

† Obesity, BMI > 30 kg/m^2^, underweight, BMI < 18.5 kg/m^2^;

** *Disseminated, positive blood culture and/or at least two non-adjacent organs affected*.

### *Candida*-reactive T cells in healthy donors and in disease control patients

In the cohort of this prospective pilot study nine disease control patients and 14 healthy donors showed no elevated frequencies of *Candida*-reactive T cells. In the cohort of 96 blood donors, the mean frequency of *C. albicans*-reactive T cells was 0.19 ± 0.11%. Combined frequency of *C. albicans*-reactive T cells among 110 healthy donors was 0.17 ± 0.12%. Mean frequencies of *C. albicans*-, *C. glabrata*-, *C. parapsilosis*-, *C. tropicalis*-, and *C. krusei*-reactive T cells in the cohort of this prospective pilot study were 0.07 ± 0.15%, 0.07 ± 0.14%, 0.05 ± 0.16%, 0.03 ± 0.05%, and 0.08 ± 0.24%, respectively.

### *Candida*-reactive T cells in patients with proven invasive *Candida* infection

We determined cut-off values to discriminate between healthy donors, disease control patients and patients with ICI regarding x-fold increase of *Candida*-reactive T cells and frequencies of *Candida*-reactive T cells using ROC-analysis. Measurements of healthy donors and disease controls were defined negative (combined cohort of blood donors, healthy donors and disease controls), proven invasive *Candida* infection was defined as a positive test. A cut-off value of 3.05-fold increase of antigen stimulated CD69^+^/CD154^+^ T cells compared to the unstimulated CD4^+^ T cells was calculated to discriminate best between individuals with and without ICI and to assure a sufficient stimulation (Figure S1). Test positivity considered elevated levels of *Candida*-reactive T cells with simultaneously 3.05-fold increase of antigen-stimulated T cells compared to unstimulated T cells to exclude false-positive results. Cut-off values for elevated levels of *C. albicans*-reactive T cells were 0.40% CD69^+^/CD154^+^ T cells among CD4^+^ T cells, for *C. glabrata* 0.22% and for *C. parapsilosis* 0.40%, respectively. As no patients with invasive *C. tropicalis or C. krusei* infections were in our cohort, we used the cut-off value of pooled *Candida* spp.*-*reactive T cells of 0.40% as cut-off value for *C. tropicalis* and *C. krusei* instead (Figure S1).

### Sensitivity and specificity of *Candida*-reactive T cells

**Figure 3:**
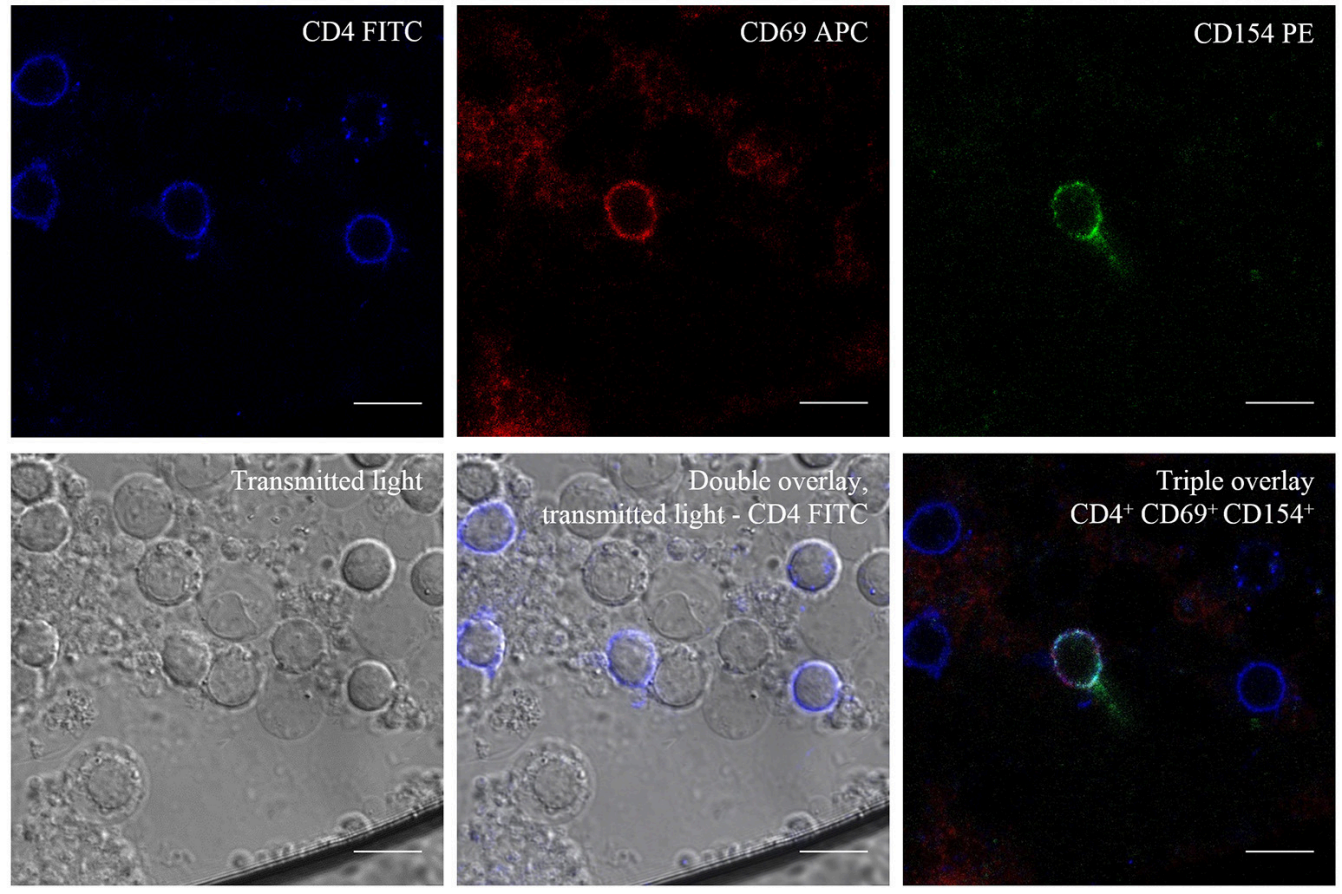
Transmitted Light—Differential Interference Contrast (DIC) and Fluorescence Microscopy of *C. albicans*-reactive T cells. CD4^+^ T cells after antigen stimulation with *C. albicans* lysate and show triple positivity, CD4^+^/CD69^+^/CD154^+^ after stimulation. Scale bar = 10 μm.

*Candida*-reactive CD4^+^ T cells were detected based on the upregulation of the activation markers CD69 and CD154 (CD40L) (Figures 1, 3). We detected elevated levels of *Candida*-reactive T cells in 13/16 patients (81.3%) with proven ICI. In three patients (18.7%) with proven ICI no values could be determined due to autofluorescence and these were excluded from this study. In 23 individuals without ICI there were no elevated levels of *Candida*-reactive T cells. The sensitivity and specificity in our study were 81.3% and 100%, respectively (Table S1). This results in positive and negative predictive values of 100% and 88.5%. The correlation between elevated levels of *Candida*-reactive T cells and the clinical diagnosis of proven ICI was statistically significant with a *p* < 0.001 (Table S1). When excluding autofluorescent patients from analysis sensitivity increased to 100% with a specificity of 100% (Table S2).

**Figure 4:**
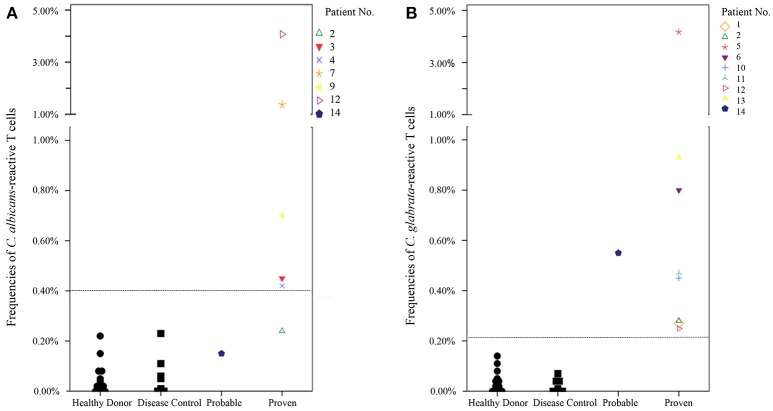
Frequencies of *Candida*-reactive T cells in healthy donors, disease control and patients with probable or proven invasive *Candida* infection. Patients with proven invasive *Candida* infection had a simultaneous 3.05-fold increase of antigen-stimulated T cells compared to unstimulated T cells to exclude false positive results and to determine quality of the stimulation. Dashed lines show cut-off values for *C. albicans*-reactive T cells (0.40%) and *C. glabrata*-reactive T cells (0.22%), respectively. Healthy donor *n* = 14, disease control *n* = 9. **(A)** Frequencies of *C. albicans* CD69^+^/CD154^+^ T cells among CD4^+^ T cells in donor/patient cohorts. Given is the highest frequency during the test series. **(B)** Frequencies of *C. glabrata* CD69^+^/CD154^+^ T cells among CD4^+^ T cells in donor/patient cohorts. Given is the highest frequency during the test series.

In 10/12 patients (83.3%) with proven ICI the *Candida*-reactive lymphocyte assay identified the same *Candida* spp. causing ICI as standard diagnostics (Table 2). Frequencies of *C. albicans*-reactive T cells and *C. glabrata*-reactive T cells in healthy donors, disease control and patients with probable or proven ICI are given in Figure 4. In one patient with a *C. albicans* candidemia and endophthalmitis we found elevated frequencies of *C. glabrata*-reactive T cells instead. In another patient with a *C. albicans* candidemia we measured *C. albicans-* and *C. parapsilosis*-reactive T cells. We determined elevated levels of *C. albicans, C. glabrata, C. parapsilosis*, and *C. tropicalis* in a histologically proven case with hepatosplenic candidiasis without species identification and we excluded this patient from contingency analysis (Table 2). This leads to a sensitivity and a specificity of the *Candida*-reactive lymphocyte assay identifying ICI and the causing *Candida* spp. among evaluable patients with proven ICI of 83.3 and 100%, respectively. The positive and negative predictive values to detect ICI and the causing *Candida* spp. by species level were 100 and 92%. The *p*-value was < 0.001 calculated by Fisher's exact test (Table S3).

**Table 2:** Patients with proven and probable invasive *Candida* infections and corresponding frequencies of *Candida*-reactive T cells.

					**Direct fungal evidence**				
**Patient**	**Underlying disease/Host factor**	**Risk factor**	**Radiological findings**	**Beta-D-Glucan**	**Histology**	**Fungal culture**	**Invasive *Candida* Infection**	**EORTC grade**	**Increased T cell frequency**	**Cross-reactivity**
1	Chronic kidney disease Mesenteric ischemia	CVC Dialysis ICU Parenteral nutrition Surgery	Neg.	N.D.	N.D.	BC and TC (Peritoneum): *C. glabrata* BC: *C. parapsilosis*	Candidemia Peritonitis	Proven	*C. glabrata C. parapsilosis*	Neg.
2	Diabetes mellitus Pancreatic carcinoma	CVC ICU Surgery	Neg.	N.D.	N.D.	BC: *C. albicans*	Candidemia Endophthalmitis	Proven	*C. glabrata*	Neg.
3	Urothelial carcinoma	Surgery	Neg.	N.D.	N.D.	BC: *C. albicans*	Candidemia	Proven	*C. albicans*	Neg.
4	Chronic liver disease Colorectal carcinoma Obesity	Chemotherapy Surgery	Neg.	N.D.	N.D.	BC: *C. albicans*	Candidemia	Proven	*C. albicans C. parapsilosis*	Pos.
5	Pancreatic carcinoma Obesity	CVC Dialysis ICU Surgery	Abdominal CT	N.D.	N.D.	BC: *C. glabrata; C. parapsilosis*	Candidemia Hepatosplenic Candidiasis	Proven	*C. glabrata C. parapsilosis*	Neg.
6	Chronic liver disease Pancreatic carcinoma	Chemotherapy CVC Radiotherapy Surgery	N.D.	N.D.	N.D.	BC: *C glabrata*	Candidemia	Proven	*C. glabrata*	Neg.
7^*^	Burkitt-Lymphoma HIV/AIDS Rheumatic disease	Chemotherapy CVC ICU Neutropenia	Spinal PET/CT Spinal MRI Spinal CT	Neg.	Neg.	BC: *C. albicans* TC: (Spinal disc and psoas abscess): *C. albicans*	Candidemia Osteomyelitis Spondylodiscitis	Proven	*C. albicans*	Neg.
8	Diabetes mellitus Hodgkin Lymphoma Obesity Viral pneumonia	Chemotherapy CVC ICU Surgery	Neg.	N.D.	N.D.	BC: *C. albicans*	Candidemia	Proven	*C. albicans*	Neg.
9	HIV/AIDS Non-Hodgkin Lymphoma Underweight	Chemotherapy CVC ICU Radiotherapy	Neg.	N.D.	N.D.	BC and CVC culture: *C. albicans*	Catheter related bloodstream infection Candidemia	Proven	*C. albicans*	Neg.
10	Alcohol addiction AML	Chemotherapy CVC ICU Neutropenia Surgery	Neg.	N.D.	N.D.	BC: *C. glabrata*	Candidemia	Proven	*C. glabrata*	Neg.
11	Alcohol addiction Chronic liver disease Diabetes mellitus Underweight	CVC Parental nutrition	Neg.	N.D.	N.D.	BC: *C. glabrata*	Candidemia	Proven	*C. glabrata*	Neg.
12	ALL	Chemotherapy	Abdominal CT, Abdominal MRI, Abdominal Ultrasound	Pos.	Pos.	Neg.	Hepatosplenic Candidiasis	Proven	*C. albicans, C. glabrata C. parapsilosis, C. tropicalis*	Pos.^†^
13	Chronic renal disease Diabetes mellitus Obesity	CVC Dialysis ICU	Neg.	N.D.	N.D.	BC: *C. glabrata*	Candidemia	Proven	*C. glabrata*	Neg.
14	AML	Chemotherapy Neutropenia	Abdominal Ultrasound	Pos.	Neg.	Neg.	Hepatosplenic Candidiasis	Probable	*C. glabrata*	Neg.

Classification of invasive Candida infections according to the 2008 EORTC/MSG criteria, culture or histology of Candida spp. and Candida-reactive T cells (De Pauw et al., 2008). Pos., positive; Neg., negative; N.D., not done; AIDS, acquired immunodeficiency syndrome; ALL, Acute lymphatic leukemia; AML, Acute myeloid leukemia; BC, blood culture; CT, Computer Tomography; CVC, central venous catheter; HIV, human immunodeficiency virus; ICU, intensive care unit; MRI, Magnetic Resonance Imaging; PET, Positron Emission Tomography; TC, tissue culture;

* *(Koehler et al., 2017)*.

† *Candida spp. causing hepatosplenic candidiasis have not been identified, cross-reactivity may represent mixed infection*.

## Discussion

In this pilot study, we evaluated the performance of a new *Candida*-reactive lymphocyte assay as diagnostic tool for ICI. Elevated levels of *Candida*-reactive CD4^+^ T cells were measured based on the upregulation of the activation markers CD69 and CD154 (CD40L) in patients with proven and probable ICI (Frentsch et al., 2005; Bacher et al., 2013, 2014, 2015; Cossarizza et al., 2017). Identification to species level was in agreement with conventional diagnostics in 83.3% of proven ICI. Disease controls and healthy controls had no elevated T cell count. In this cohort, there was negligible cross-reactivity between the different *Candida* spp. causing ICI.

The *Candida*-reactive lymphocyte assay compares favorable characteristics to current gold-standard diagnostic procedures. Volatile sensitivity ratios of blood culture are caused by the rapid elimination of viable *Candida* cells from blood circulation (Cuenca-Estrella et al., 2012; Nguyen et al., 2012; Kullberg and Arendrup, 2015; Pappas et al., 2016). The *Candida*-reactive lymphocyte assay may improve sensitivity of and time to the diagnosis of ICI including identification of the causative pathogen to species level and could therefore considerably improve the treatment of patients with ICI. However, both blood and tissue culture currently remain the only diagnostics allowing for susceptibility testing. Microscopy, histopathology, and culture of infectious tissue samples require surgery or other invasive procedures with significant risks for the patient, whereas the *Candida*-reactive lymphocyte assay is a non-invasive, peripheral blood test with little to no risks for the patient (Clancy and Nguyen, 2013).*Candida* mannan antigen and antimannan antibodies, CAGTA and BDG-measurements do not permit discrimination between different *Candida* spp. and therefore in contrast to the *Candida*-reactive lymphocyte assay they do not allow for definitive diagnosis and tailored treatment (Laín et al., 2007; Kullberg and Arendrup, 2015).

As reported previously, we were able to provide flow-cytometry based diagnostics with *Candida*-reactive T cells for invasive candidiasis in a case of a patient with a human immunodeficiency virus (HIV)-associated Burkitt lymphoma with a *C. albicans* spondylodiscitis (Koehler et al., 2017). We were able to diagnose ICI by species level, whereas in treatment course blood culture remained negative (Table 2) (Koehler et al., 2017). In this case, the *Candida*-reactive lymphocyte assay favored conventional diagnostics due to improved sensitivity and shortened time to diagnosis (Koehler et al., 2017).

Measuring antigen-reactive T cells by the upregulation of the activation markers CD69 and CD154 (CD40L) can be expanded to other pathogens with challenging diagnosis such as *Aspergillus* spp. and *Mucorales* spp. (Bacher et al., 2013, 2014, 2015; Potenza et al., 2016; Cossarizza et al., 2017). However, it remains unknown, how the *Candida*-reactive lymphocyte assay performs when dealing with other *Candida* spp. causing ICI than *C. albicans, C. glabrata, C. parapsilosis, C. tropicalis*, or *C. krusei*.

Limitations of the *Candida*-reactive lymphocyte assay are insufficient cell count of T cells or lack of antigen-presenting cells, as well as autofluorescence of patient cells with elevated background levels of CD69 and CD154. Autofluorescence of cells is mainly caused by dead cells, which bind antibodies in a low-affinity and unspecific manner (Cossarizza et al., 2017). To avoid high yields of autofluorescent cells, dead cell staining, as well as processing and cultivation of PBMC without delay remain mandatory (Cossarizza et al., 2017; Wurster et al., 2017).

The performance of the *Candida*-reactive lymphocyte assay may be improved by the combined determination of activation markers and cytokines, which are typically secreted by reactive CD4^+^ T cells upon activation such as interleukin-17, interleukin-22 or interferon-γ (Potenza et al., 2016; Cossarizza et al., 2017).

With the reduction of fungal load by antimycotic treatment or even surgery in case of invasive mold infection, we expect the levels of fungus-reactive T cells to be decreasing over time, which may affect the performance of the assays in patients with extensive treatment in the past medical history, but it also provides a simple and sensitive opportunity to follow treatment courses of patients (Bacher et al., 2015). In our cohort, low number of proven and probable ICI patients may hamper the strength of the results of this pilot project so that further studies with larger patient numbers remain mandatory also analyzing the course of *Candida*-reactive T cells over the sequence of therapy.

In summary, the *Candida*-reactive lymphocyte assay may complement current diagnostics for ICI, especially when blood cultures remain negative, as well as in cases of underlying medical conditions with contraindication for biopsies or surgery. The *Candida*-reactive lymphocyte assay is a non-invasive, peripheral blood test and it allows the identification of *Candida* spp. for targeted treatment. As the *Candida*-reactive lymphocyte assay may establish the diagnosis of ICI within 36–48 h, it may enable earlier treatment in case of prolonged cultivation of blood cultures.

## Author contributions

FK performed experiments, contributed collection and assembly of data, data analysis and interpretation, manuscript writing and final manuscript approval. OC conceived the study idea, contributed collection and assembly of data, data analysis and interpretation, manuscript writing and final manuscript approval. HW contributed *Candida* strains for mechanical lysis, BDG-data, data analysis and interpretation, manuscript writing and final manuscript approval. ACS and JS-G contributed data analysis and interpretation, manuscript writing, and final manuscript approval. HO, PB, and AS contributed collection and assembly of data, data analysis and interpretation, manuscript writing and final manuscript approval. MZ, RA, and AR contributed data analysis and interpretation, manuscript writing and final manuscript approval. PK conceived the study idea, performed experiments, contributed collection and assembly of data, data analysis and interpretation, manuscript writing and final manuscript approval.

### Conflict of interest statement

FK reports grants from the German Federal Ministry of Research and Education, non-financial support from Miltenyi Biotec GmbH during the conduct of the study. OC reports research grants from Actelion, Amplyx, Arsanis, Astellas, AstraZeneca, Basilea, Bayer, Cidara, Duke University, F2G, Gilead, GSK, Leeds University, Matinas, Medicines Company, MedPace, Melinta, Merck/MSD, Miltenyi Biotec GmbH, Pfizer, Rempex, Roche, Sanofi Pasteur, Scynexis, Seres, is a consultant to Amplyx, Actelion, Astellas, Basilea, Cidara, Da Volterra, F2G, Gilead, Janssen, Matinas, Menarini, Merck/MSD, Paratek, PSI, Scynexis, Seres, Summit, Tetraphase, Vical, and received lecture honoraria from Astellas, Basilea, Gilead, Merck/MSD and Pfizer outside the submitted work. HW reports personal fees and non-financial support from BeckmanCoulter, non-financial support from Specific Technologies, Accelerate and Cepheid outside the submitted work. HO reports personal fees from Astra Zeneca, Bayer, BMS, Gilead, Leo, MSD, Orphan, and Pfizer outside the submitted work. AS reports other from Miltenyi Biotec GmbH outside the submitted work. RA and AR reports personal fees from Miltenyi Biotec GmbH during the cnduct of the study. PK reports indirect personal fees from the Cologne Cluster of Excellence—Cellular Stress Responses in Aging-Associated Diseases (CECAD), University of Cologne as CECAD rotational “Gerok” position, non-financial support from Miltenyi Biotec GmbH during the conduct of the study; non-financial support from Merck/MSD and MedImmune and lecture honoraria from Astellas outside the submitted work. The remaining authors declare that the research was conducted in the absence of any commercial or financial relationships that could be construed as a potential conflict of interest.

## Abbreviations

ICI: Invasive *Candida* Infection
CAGTA: *Candida albicans* Germ Tube Antibody
BDG, β-1: 3-D-glucan
PCR: Polymerase Chain Reaction
ISI: Improving Diagnosis of Severe Infections of Immunocompromised Patients
EDTA: Ethylenediaminetetraacetic acid
7AAD: 7-aminoactinomycin D
SEB: Staphylococcal Enterotoxin B
ID: Species identification
AST: Susceptibility testing
ECMM: European Confederation of Medical Mycology
EORTC/MSG: European Organization for Research and Treatment of Cancer/Invasive Fungal Infections Cooperative Group and the National Institute of Allergy and Infectious Diseases Mycoses Study Group
ROC: Receiver operating characteristic analysis
ICU: Intensive care unit
HIV: Human immunodeficiency virus.
