# Supplementary material for: Candida-Reactive T Cells for the Diagnosis of Invasive Candida Infection—A Prospective Pilot Study
Source: Front Microbiol. 2018 Jun 22;9:1381. doi: 10.3389/fmicb.2018.01381 (PMC6024001; doi:10.3389/fmicb.2018.01381)
Supplement: Supplementary file 1 [file Image_1.pdf]

Figure S1. Cut-off determination.

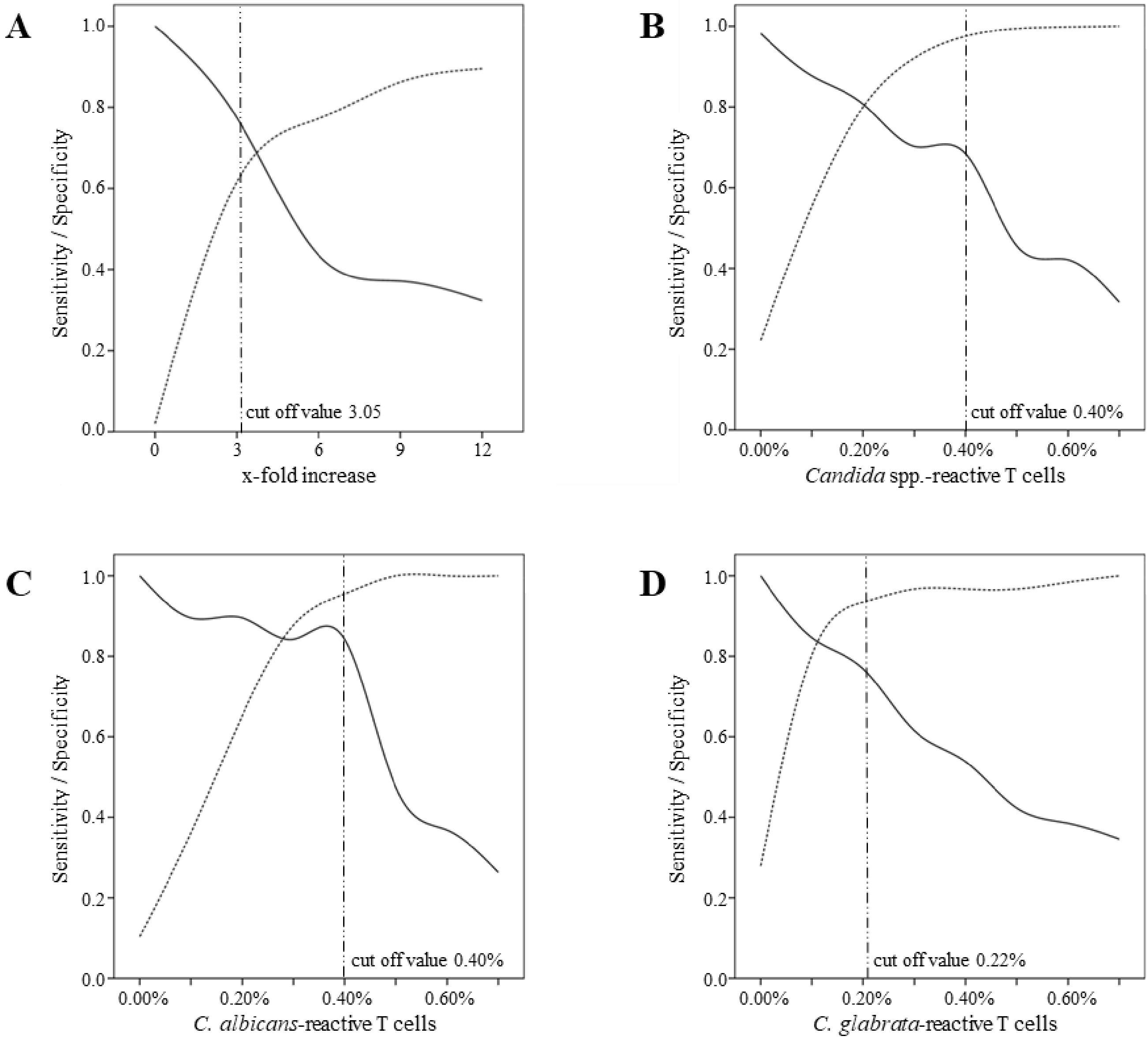

Cut-off values to discriminate between healthy donors and disease control and patients with proven invasive *Candida* infection and were calculated by receiver operating characteristic (ROC) curve analysis. Given is the fold increase and the frequency of *Candida*-reactive $CD69^{+}/CD154^{+}$ T cells among $CD4^{+}$ T cells with the respective sensitivity and specificity. Solid line = sensitivity, dashed line = specificity. (**A**) Cut-off determination of fold-increase of *Candida*-reactive T cells compared to unstimulated $CD4^{+}$ T cells. 456 measurements of 119 individuals without invasive *Candida* infection; 62 measurements of 13 individuals with proven invasive *Candida* infection. (**B**) Cut-off determination of elevated levels of *Candida spp*. $CD69^{+}/CD154^{+}$-reactive T cells among $CD4^{+}$ T cells. 483 measurements of 119 individuals without invasive *Candida* infection; 57 measurements of 13 individuals with proven invasive *Candida* infection. (**C**) Cut-off determination of elevated levels of *C. albicans*-reactive T cells $CD69^{+}/CD154^{+}$-reactive T cells among $CD4^{+}$ T cells. 155 measurements of 127

individuals without invasive *C. albicans* infection; 19 measurements of 6 individuals with proven invasive *C. albicans* infection. (**D**) Cut-off determination of elevated levels of *C. glabrata*-reactive T cells $CD69^+/CD154^+$-reactive T cells among $CD4^+$ T cells. 61 measurements of 31 individuals without invasive *C. glabrata* infection; 26 measurements of 6 individuals with proven invasive *C. glabrata* infection.
