# Supplementary material for: Candida-Reactive T Cells for the Diagnosis of Invasive Candida Infection—A Prospective Pilot Study
Source: Front Microbiol. 2018 Jun 22;9:1381. doi: 10.3389/fmicb.2018.01381 (PMC6024001; doi:10.3389/fmicb.2018.01381)
Supplement: Supplementary file 2 [file Table_1.DOCX]

**Table S1. Contingency table of all analyzed patients, excluded patient with probable invasive *Candida* infection.**

| **Diagnosis of invasive *Candida* infection according to EORTC/MSG consensus** | ***Candida*-reactive T-cell frequencies** | | **Total patient number** |
| --- | --- | --- | --- |
|  | **Increased** | **Not increased** |  |
| **Proven** | 13 | 3 | 16 |
| **No established diagnosis of ICI** | 0 | 23 | 23 |
| **Total** | 13 | 26 | 39 |

p < 0.001 by Fisher’s exact test, sensitivity (95% confidence interval) = 81.3% (54.4% - 96.0%), specificity (95% confidence interval) = 100% (85.2% - 100.0%).
