# Supplementary material for: Candida-Reactive T Cells for the Diagnosis of Invasive Candida Infection—A Prospective Pilot Study
Source: Front Microbiol. 2018 Jun 22;9:1381. doi: 10.3389/fmicb.2018.01381 (PMC6024001; doi:10.3389/fmicb.2018.01381)
Supplement: Supplementary file 4 [file Table_3.DOCX]

**Table S3. Contingency table of matching *Candida* spp. causing invasive *Candida* infection identified by the *Candida*-reactive T cell assay and by culture in patients with evaluable T cell frequencies.** Patient with proven invasive *Candida* infection and missing identification of *Candida spp*. causing invasive *Candida* infection and patient with probable invasive *Candida* infection excluded.

| **Diagnosis of invasive *Candida* infection according to EORTC/MSG consensus** | ***Matching Candida* spp. identified by the *Candida*-reactive T-cell assay and by culture** | | **Total patient number** |
| --- | --- | --- | --- |
|  | **match** | **No match** |  |
| **Proven** | 10 | 2 | 12 |
| **No established diagnosis of ICI** | 0 | 23 | 23 |
| **Total** | 10 | 25 | 35 |

p < 0.001 by Fisher’s exact test, sensitivity (95% confidence interval) = 83.3% (51.6% - 97.9%), specificity (95% confidence interval) = 100% (85.2% - 100.0%).
